# Early Bio-Efficacy Loss of Nets Mass Distributed for Malaria Vector Control in Madagascar in 2018: Implications for Malaria Prevention

**DOI:** 10.4269/ajtmh.24-0858

**Published:** 2025-10-23

**Authors:** Thiery Nepomichene, Rico Randrenjarison, Jacky Raharinjatovo, Isabel Swamidoss, Carla Mapp, Laurent Kapesa, Jocelyn Razafindrakoto, Anna Bowen, Allison Belemvire, Sarah Zohdy, Stephen Poyer, Romain Girod

**Affiliations:** ^1^ Medical Entomology Unit, Institut Pasteur de Madagascar, Antananarivo, Madagascar;; ^2^ Population Services International Madagascar, Antananarivo, Madagascar;; ^3^ Malaria Branch, US President’s Malaria Initiative, US Centers for Disease Control and Prevention, Atlanta, Georgia;; ^4^ US President’s Malaria Initiative, USAID, Antananarivo, Madagascar;; ^5^ US President’s Malaria Initiative, US Centers for Disease Control and Prevention, Antananarivo, Madagascar;; ^6^ US President’s Malaria Initiative, USAID, Washington, District of Columbia;; ^7^ Entomology Branch, US President’s Malaria Initiative, US Centers for Disease Control and Prevention, Atlanta, Georgia;; ^8^ Population Services International, Washington, District of Columbia;; ^9^ Institut Pasteur, Paris, France

## Abstract

In 2018, insecticide-treated nets (ITNs) were mass distributed across Madagascar. The bio-efficacy of DawaPlus^®^ 2.0 and PermaNet^®^ 2.0 ITNs was assessed upon arrival and at 12, 24, and 36 months after distribution. Chemical analyses of insecticide residue on ITNs were also conducted. On arrival, mosquito mortality rates observed when exposed to DawaPlus 2.0 (86.4%) and PermaNet 2.0 nets (83.6%) exceeded the WHO’s threshold of 80.0%. At 12, 24, and 36 months after distribution, mosquito mortality rates were <56% for all districts. Moreover, the knockdown effect was below the WHO threshold of 95.0% for all districts and at all time points, even for new ITNs. With the exception of the new DawaPlus 2.0, the deltamethrin residue on ITNs was also lower than the expected ranges of 80 mg/m^2^ ± 25% for DawaPlus 2.0 and 55 mg/m^2^ ± 25% for PermaNet 2.0; regardless of ITN age, the concentration of deltamethrin was <66 mg/m^2^ for DawaPlus 2.0 and <36 mg/m^2^ for PermaNet 2.0 ITNs. According to the manufacturers, ITNs are effective for 36 months; therefore, mass distribution campaigns are organized every 3 years. However, the DawaPlus 2.0 and PermaNet 2.0 ITNs exhibited a loss of bio-efficacy within 1 year of distribution. This bio-efficacy loss could be due to a manufacturing problem, poor storage and transportation conditions, or poor use and net care practices in Madagascar. Understanding and correcting the root causes of this issue is critical for guiding corrective actions, such as improving manufacturing processes, replacing ITNs more frequently, and increasing education on ITN care.

## INTRODUCTION

In Madagascar, malaria remains a serious public health concern, primarily in remote rural areas. In 2017, Madagascar was among the 10 countries in Africa with the highest malaria burden that reported increases in cases compared with 2016.^1^ The decline in malaria cases and deaths globally has slowed and now appears to be stalled. Various causes may be contributing factors, including the failure of vector control tools. One key malaria vector control strategy is based on insecticide-treated nets (ITNs), which are typically mass distributed every 3 years. Insecticide-treated nets contain at least one insecticide within the pyrethroid class, such as deltamethrin, alpha-cypermethrin, or permethrin. They are made of polyester or polyethylene materials, depending on the manufacturer, and the insecticide is either applied as a coating on the textile or incorporated into the fibers. Until the end of 2018, 14 prequalified ITN products were listed on the WHO Vector Control Product List. The insecticide is expected to last for 3 years according to WHO recommendations and manufacturer specifications. However, loss of net bio-efficacy before the expected duration has been reported in the Democratic Republic of Congo and India.^2^^,^^3^

The bio-efficacy of nets is assessed by measuring the degree of knockdown and mortality induced by insecticide in susceptible mosquitoes, as determined via the WHO cone bioassay, or the inhibition rate of blood feeding of susceptible mosquitoes, as determined using the WHO tunnel test. Insecticide on nets provides additional protection for net users and the community when ITN coverage is high.^2^ Mosquitoes can be killed or their blood-feeding altered, which reduces the chance of malaria transmission among both ITN users and nonusers.^3^ Insecticide on nets could also reduce the density of adult mosquitoes, in turn decreasing community exposure to vector bites.^4^^,^^5^ Changes in vector age structure, with mosquito populations becoming younger and parasites unable to complete their full development before mosquito death, have also been demonstrated. Even in the presence of insecticide-resistant vectors, ITNs can provide protection: in addition to providing a physical barrier, ITNs with sublethal levels of pyrethroid can reduce vector fitness.^5^

In Madagascar, in 2017, with 62.1% of the population having access to ITNs, the ITN served as the primary vector control tool.^1^ In September 2018, the US President’s Malaria Initiative procured 5,988,346 nets, branded DawaPlus^®^ 2.0 and PermaNet^®^ 2.0, intended for distribution in 57 districts, in addition to those procured by the Global Fund. As recommended by the WHO, the durability, including the bio-efficacy, of ITNs in use within households must be monitored after their distribution.

The objective for this study was to evaluate the bio-efficacy and insecticide residuals of DawaPlus 2.0 and PermaNet 2.0 nets before distribution and at 12, 24, and 36 months post-distribution, using the standard WHO cone test and high-performance liquid chromatography.

## MATERIALS AND METHODS

### Insecticide-treated net sampling.

DawaPlus 2.0 nets are manufactured by Tana Netting (Bangkok, Thailand). They are made of a 100-denier white polyester textile coated with an 80 mg/m^2^ loading dose of deltamethrin and obtained an interim WHO Pesticide Evaluation Scheme (WHOPES) recommendation in July 2009.^6^ PermaNet 2.0 nets are manufactured by Vestergaard–Frandsen (Lausanne, Switzerland). They are made of a 100-denier polyester, multifilament textile treated to a target concentration of 55 mg/m^2^ of deltamethrin^7^ and received a complete WHOPES recommendation in 2008. The WHOPES has since transitioned to Prequalified Vector Control Products.

**Figure 1.**
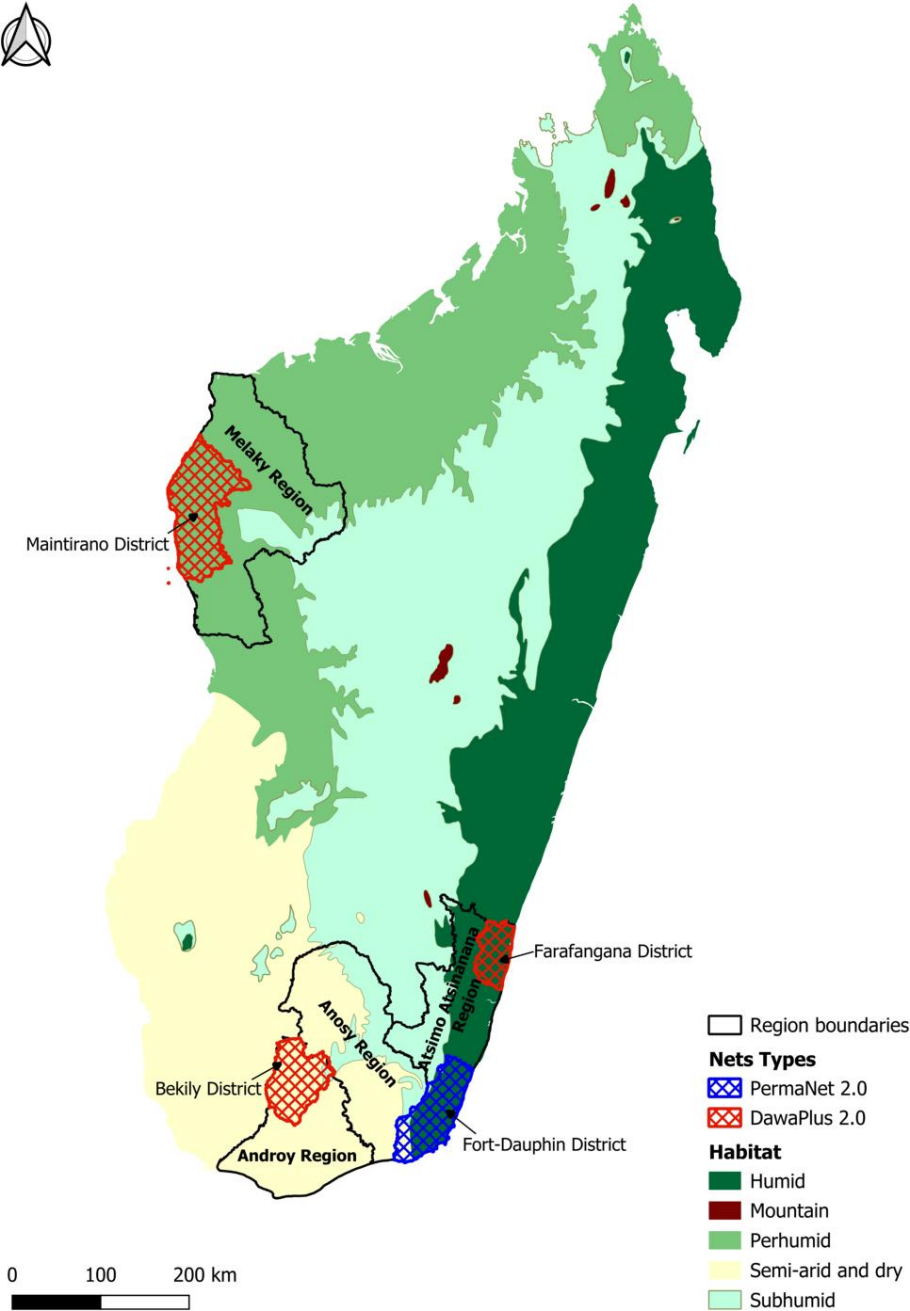
Locations of net sampling.

DawaPlus 2.0 nets were evaluated before distribution (as new, packaged nets) and subsequently at 1, 12, 24, and 36 months post-distribution. In contrast, bio-efficacy testing of PermaNet 2.0 nets commenced at 12 months post-distribution and continued at 24 and 36 months. DawaPlus 2.0 nets were collected from three districts in Madagascar with differing ecosystems: Bekily (semi-arid), Farafangana (humid), and Maintirano (tropical), whereas PermaNet 2.0 nets originated from the sub-humid Fort Dauphin District (Figure 1).

At each study time point, 30 ITNs of each brand were withdrawn for bioassay testing and chemical residue analysis at each study site. However, at the baseline round, only 22 ITNs were collected from the Bekily and Maintirano Districts, with none retrieved from Fort Dauphin (without a baseline round). During the baseline, 12-month, and 24-month surveys, ITNs for bioassay and chemical analyses were obtained from neighboring households rather than being withdrawn from cohort households; in each case, the collected ITNs were replaced with new ones. At the study endline, cohort ITNs were collected from study households as the longitudinal assessment concluded during the 36-month round. Households participating in the bioassay received a replacement ITN of the same brand. Bioassays were conducted on 22 to 30 DawaPlus 2.0 and 30 PermaNet 2.0 nets per time point and district.

### Insecticide-treated net bioassay processing.

For each selected ITN, a 25″ × 25″ subsample was cut from each of the four sides and the roof and placed in an aluminum foil envelope, labeled, and kept individually in a +4°C refrigerator before the bioassay was conducted. Standard WHO cone bioassays were performed as recommended by the WHOPES.^8^ A fully insecticide-susceptible laboratory-confirmed mosquito strain of *Anopheles arabiensis* (*An. arabiensis*) was used.^9^

For each subsample, four cone tests were conducted simultaneously, in accordance with the standard WHO procedure.^8^ Five non-blood-fed, 2- to 5-day-old female mosquitoes were introduced into each cone and exposed to ITN subsamples for 3 minutes before being transferred to paper cups covered with neutral netting. The mosquitoes were then held for 24 hours at 25°C ± 2°C and 70% ± 5% humidity with access to a 10% sugar solution.

The knockdown (KD) rate was recorded 60 minutes post-exposure, and the mortality rate was recorded 24 hours post-exposure. Following this methodology, each net was tested with a total of 100 mosquitoes, encompassing all four sides and the roof of the enclosure. On each day of testing, four cones, each containing 10 *An. arabiensis* mosquitoes, were fixed on a nonimpregnated net as a negative control. If the mortality rate in the control was <10% for a given day, the data were adjusted using Abbott’s formula. If the mortality rate in the control was >10%, all the tests for that day were repeated. An ITN was considered effective if the mortality rate met the WHO threshold of ≥80% or the KD rate met the WHO threshold of ≥95%.^8^

### Chemical residue analysis.

Chemical analyses of 10 nets randomly selected from the 22 to 30 ITNs bioassayed per brand and per district and time point were conducted at the US CDC in Atlanta, Georgia. Five pieces of netting were tested per net, including samples from all four sides and the roof, taken from the same locations as those used for bioassays. The deltamethrin R-alpha isomer content was determined using the collaborative international pesticides analytical council method 333/LN/(M)/3, which involves extraction by sonication and shaking with an isooctane/dioxane mixture (80/20, v/v), followed by chromatographic determination via high-performance liquid chromatography with ultraviolet diode array detection.

## RESULTS

The average KD effect was below the WHO threshold of 95.0% for both DawaPlus 2.0 and PermaNet 2.0 nets in all districts and time points. Before distribution, the KD effects of both DawaPlus 2.0 and PermaNet 2.0 nets were 79.3% and 71.2%, respectively. The KD value exceeded the WHO threshold for only three (of 30; 10.0%) DawaPlus 2.0 nets and two (of 30; 6.67%) PermaNet 2.0 nets. One month after distribution, the KD rates were 66.7%, 65.6%, and 66.4% for the Bekily, Farafangana, and Maintirano Districts, respectively, for DawaPlus 2.0 nets. At 12 months post-distribution, the average KD values were 58.3%, 62.6%, and 65.1% for DawaPlus 2.0 nets from the Bekily, Farafangana, and Maintirano Districts, respectively, and 53.7% for PermaNet 2.0 nets from the Fort Dauphin District. At 24 months post-distribution, the average KD values were 45.8%, 31.2%, and 36.8% for DawaPlus 2.0 nets from the Bekily, Farafangana, and Maintirano Districts, respectively, and 49.9% for PermaNet 2.0 nets from the Fort Dauphin District. Finally, 36 months post-distribution, the average KD values were 89.9%, 81.7%, and 87.5% for DawaPlus 2.0 nets from the Bekily, Farafangana, and Maintirano Districts, respectively, and 93.5% for PermaNet 2.0 nets from the Fort Dauphin District.

**Figure 2.**
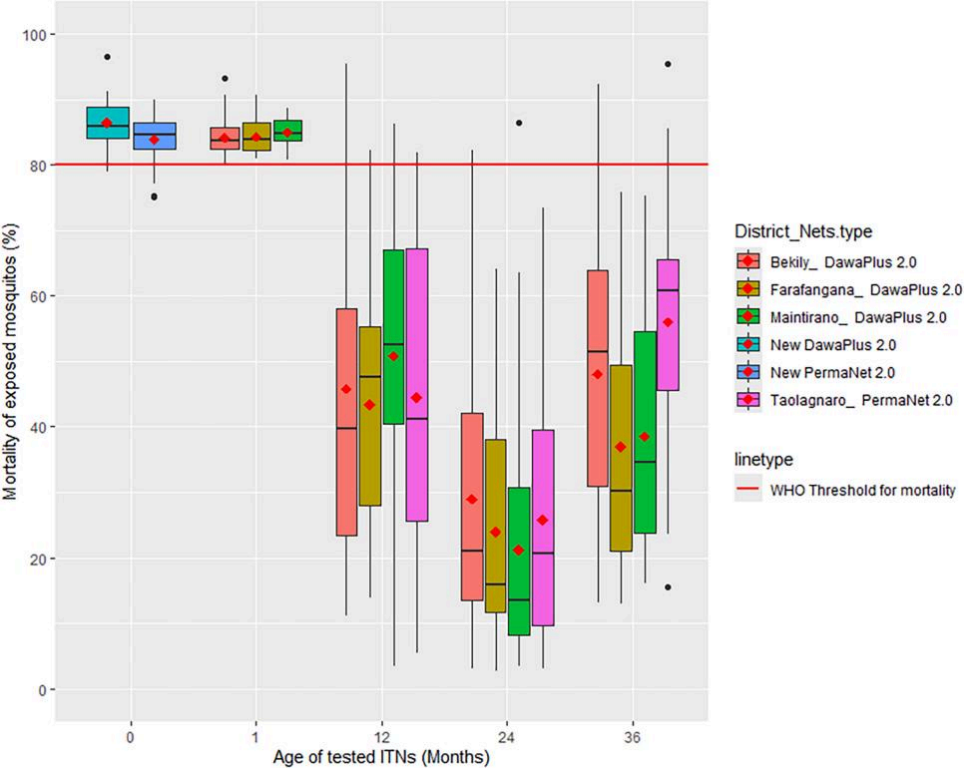
*Anopheles arabiensis* mortality induced by DawaPlus^®^ 2.0 and PermaNet^®^ 2.0 nets mass distributed in Madagascar in 2018.

In contrast, before distribution, DawaPlus 2.0 and PermaNet 2.0 nets induced 86.4% and 83.9% mortality rates, respectively, which exceeded the WHO threshold (80.0%). After 1 month, mortality rates induced by DawaPlus 2.0 nets were 84.0%, 84.5%, and 84.9% for the Bekily, Farafangana, and Maintirano Districts, respectively, which also exceeded the WHO threshold. At 12, 24, and 36 months post-distribution, the average mosquito mortality rates induced by both DawaPlus 2.0 and PermaNet 2.0 nets were below 56% for all districts, falling below the WHO threshold (Figure 2).

**Figure 3.**
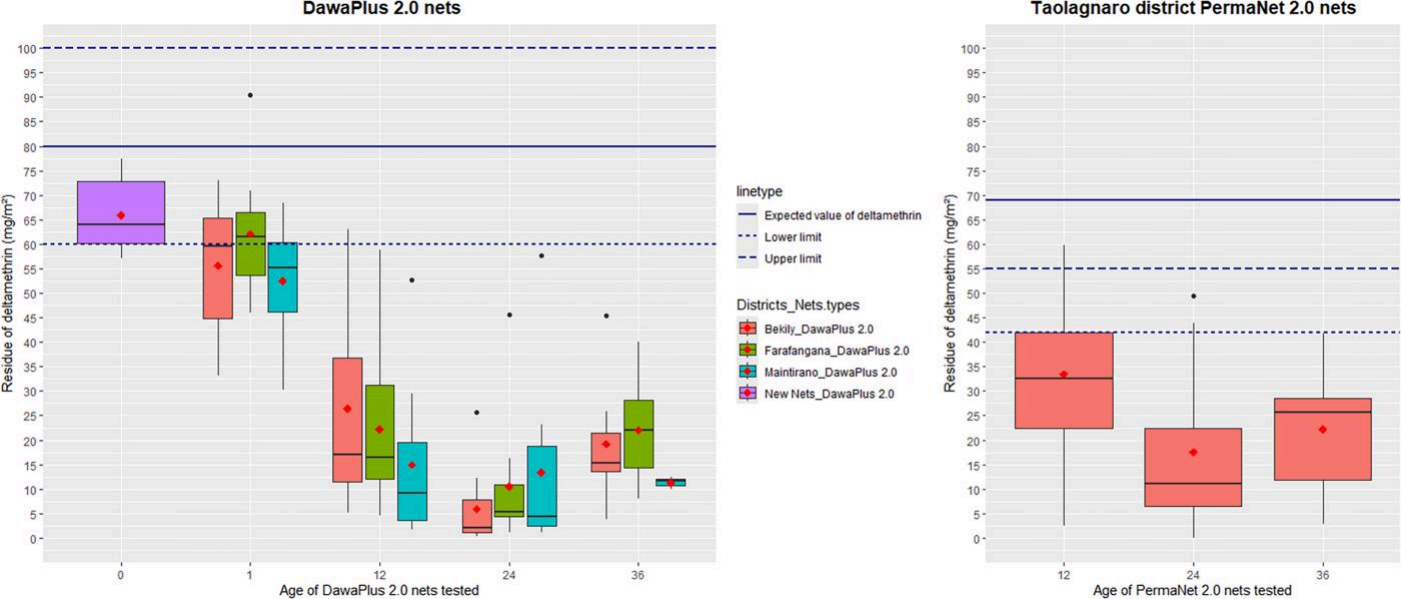
Deltamethrin residue on DawaPlus^®^ 2.0 nets mass distributed in Madagascar in 2018. DawaPlus 2.0 nets were tested before distribution and 1 month after distribution, whereas bioassays for PermaNet^®^ 2.0 nets began 12 months after distribution. Subsequently, both DawaPlus 2.0 and PermaNet 2.0 nets were bioassayed at 24 and 36 months post-distribution.

For chemical residue analysis at all-time points, the mean quantity of deltamethrin residue on each net fell below the expected values of 80 mg/m^2^ for DawaPlus 2.0 nets and 55 mg/m^2^ for PermaNet 2.0 nets (Figure 3).

## DISCUSSION

The bio-efficacy and residual chemical content of PermaNet 2.0 and DawaPlus 2.0 nets were followed for 36 months after their distribution using *An. arabiensis*, an insecticide-susceptible mosquito, according to WHO and insecticide residue analysis guidelines. The bio-efficacy of both PermaNet 2.0 and DawaPlus 2.0 nets fell below the WHO threshold within 12 months of distribution. At 36 months after distribution, an unexpected increase in KD values was observed. This may be due to the sampling of ITNs from the longitudinal cohort. As mentioned previously, the cohort ITNs were collected and tested at the study endline (during the 36-month round). These nets may have been more carefully handled by participants over time, resulting in better physical condition and retained insecticide content, which could have influenced the observed bio-efficacy results. Residual insecticide concentrations also fell below the expected range for both brands when tested 12 months after distribution. Net bio-efficacy loss decreases the protective effect of nets on users and the community, leading to an increase in malaria transmission. Mosquitoes that are not rapidly incapacitated by insecticide may make repeated feeding attempts. Moreover, the ability of ITNs to reduce entomological infection rates (the number of infective bites per person per period of time) by decreasing the density of competent vectors may be compromised in the setting of bio-efficacy loss.^2^ In Madagascar, reported malaria cases increased from ∼965,000 in 2018 and 992,000 in 2019, shortly after mass ITN distribution, to 1,950,000 in 2020 and then to 2,344,000 in 2021. Insecticide-treated nets were not redistributed via mass campaign until August to October 2021, although they were available via routine distribution channels to pregnant women and children under 5 years of age.^10^

The drivers of the observed bio-efficacy loss within 1 year of distribution were unclear. Manufacturing issues could be a source. For PermaNet 2.0 nets, a change in the polymer coating was demonstrated to impact product performance beginning in 2012, resulting in reduced bio-efficacy and wash resistance. It was also associated with increased malaria transmission in Papua New Guinea.^11^ It is not known which ones were distributed in Madagascar in 2018; however, it is likely that they were produced after 2012. Moreover, poor storage and transportation conditions could also deteriorate the nets’ performance. The temperature and humidity values were not recorded during transportation to Madagascar or during many months of storage in Madagascar before distribution. Nevertheless, the range of acceptable temperatures and humidity is wide: during up to 5 years of storage, ITNs, including DawaPlus 2.0 nets, remained efficacious against susceptible and resistant mosquito strains at temperatures ranging from 25°C to 33.4°C and relative humidity ranging from 40% to >100%.^12^ Finally, use and maintenance practices in Madagascar may not align optimally with international recommendations for net care because of limited access to mild soaps and shady places in which to dry nets. A study of net care during utilization in Madagascar should be conducted to help guide interventions aimed at improving net durability.

## CONCLUSION

Within 1 year of ITN distribution in Madagascar in 2018, both PermaNet 2.0 and DawaPlus 2.0 nets in all sampled districts had fallen below international bio-efficacy standards, putting millions of people at increased risk for malaria. Solutions, including manufacturing higher-quality ITNs, improving storage and usage conditions, and increasing the frequency of distribution, could help reduce malaria morbidity and mortality in Madagascar and more broadly in sub-Saharan Africa.
